# Use of Artificial Intelligence for the Early Detection and Prediction of Alzheimer’s Disease

**DOI:** 10.7759/cureus.109138

**Published:** 2026-05-18

**Authors:** Lhakpa T Khangsar, Andrew J Boileau

**Affiliations:** 1 Neurology, Saba University School of Medicine, The Bottom, BES; 2 Neuroscience and Neurology, Saba University School of Medicine, The Bottom, BES

**Keywords:** alzheimer’s disease, artificial intelligence, deep learning, disease progression prediction, early detection, machine learning, mild cognitive impairment, multimodal biomarkers, neuroimaging biomarkers

## Abstract

Alzheimer’s disease (AD) is a progressive neurodegenerative disorder and is a leading cause of dementia. Early detection of disease pathology and prediction of its progression from mild cognitive impairment to AD dementia has important implications in clinical practice and care planning. Current diagnostic methods largely depend on post-symptomatic clinical evaluations or invasive biomarker testing which restricts widespread adoption. This review explores six primary studies utilizing artificial intelligence techniques, specifically machine learning and deep learning algorithms trained on multimodal datasets. Studies were identified through a search of the PubMed database using outlined Medical Subject Headings (MeSH) and text-word terms, and filtered for clinical trials or randomized controlled trials. Models integrating neuroimaging, CSF biomarkers, neuropsychological assessments, and physiological signals achieved high diagnostic and predictive accuracies, surpassing traditional statistical methods. Interpretability tools improved transparency and bridged the gap between model outputs and known AD pathophysiological mechanisms. Despite significant advancements, limitations including small sample sizes, geographical and ethnic homogeneity, limited external validation, and lack of longitudinal validation persist. Future research should aim to use large-scale, multi-center, longitudinal trials to increase generalizability and build clinical trust before widespread adoption. Overall, AI shows strong potential and promise as a clinical decision support tool for early detection and predictive modeling of Alzheimer's disease.

## Introduction and background

Alzheimer’s disease (AD) is a biological construct based on the presence of abnormal core biomarkers, specifically amyloid and tau [1]. AD is a progressive neurodegenerative disorder impacting over 7 million Americans, representing 60% to 80% of dementia cases, and the fifth leading cause of death in individuals aged 65 and older in 2021. Dementia is a syndrome which includes difficulties with memory, cognition, language, and other thinking skills that affect an individual’s ability to perform everyday tasks. By 2030, all members of the baby-boom generation will be at least 65 years old, totaling around 71 million Americans and accounting for over 20% of the population. As a result of this demographic shift, improvements in mortality due to healthcare advancements, and AD’s strong association with age, the prevalence of AD is projected to rise to 13.8 million by 2060 [2].

Pathogenesis

AD is a progressive disease, meaning it gets worse with time. It is characterized by accumulations of protein fragments that clump up both inside and outside of neurons in which many researchers believe that these accumulations can lead to neuronal death. Amyloid beta (Aβ) plaques outside of neurons and abnormal tau proteins inside of neurons called tau neurofibrillary tangles (NFTs) induce neuroinflammation that cause damage to brain tissue. The Aβ plaques are made of peptides derived from amyloid precursor protein (APP) via secretase enzymes (α, β, and γ). Normal enzymatic cleavage via secretase results in Aβ1-40 soluble isoform; however, if the cleavage pattern is changed, it may result in Aβ1-42 isoform that can readily aggregate and become plaques due to the addition of two amino acids, isoleucine and alanine. This irregular cleavage is due to genetic mutations in the *APP*, *presenilin 1*, *presenilin 2*, or *apolipoprotein E* (*ApoE*) gene. Tau proteins are microtubular proteins essential in maintaining the integrity of the neuronal cytoskeleton. Mutations in the tau genes or dysregulation of kinases leads to hyperphosphorylation of tau proteins by cyclin-dependent kinase-5. This leads to NFT formation in the cytosol because of hyperphosphorylation causing a decrease in the tau protein’s affinity to microtubules. These depositions of extracellular Aβ plaques and intracellular NFTs may impair normal cellular function including synaptic communication, axonal transport, and signal transduction, leading to gradual neuronal degeneration and atrophy [3].

The pathological hallmarks of AD consist of toxic Aβ plaques and NFTs deposition causing activation of acute inflammation via Toll-like receptors in the brain. With AD’s chronic nature and persistent neuroinflammation, this results in an imbalanced release of pro- and anti-inflammatory cytokines. The proinflammatory cytokines can impair dendritic spines and disrupt microglia function resulting in ineffective removal of plaques, tangles, and cellular debris, which furthers neurodegeneration. The maximum binding of Aβ plaques is seen in the hippocampus and cortical n-acetylcholine receptors (AChRs) contributing to AD’s dementia symptoms [3]. One of the most common signs of AD is short-term memory loss like forgetting recently learned information and repeatedly asking the same questions [2]. The cholinergic neurons in the nucleus basalis of Meynert also selectively display plaques and NFTs deposition leading to symptoms of cognitive impairment, personality changes, and problems with executive function. Initial clinical symptoms of cognitive impairment include new problems with speaking and writing, challenges in planning, problem solving, difficulties completing familiar tasks, and getting lost in familiar surroundings [4].

Diagnosis

The diagnosis of AD is often challenging as it requires a combination of clinical, neuropsychological, imaging, and biomarker assessments. This can be time-consuming and costly, involves multiple providers, and is not always accurate. AD typically follows a progressive continuum that begins with asymptomatic phase (preclinical AD), where patients have biomarker evidence of AD pathology without any cognitive or behavioral decline. Patients then progress to mild cognitive or behavioral impairment (MCI/MBI), when they demonstrate impairments in language, episodic memory, executive function, and visuospatial function [5]. They are still relatively independent but symptoms vary widely at this stage so sometimes are misinterpreted as normal aging process. Patients finally develop AD dementia when they experience severe cognitive deficits along with decline in activities of daily living, immobility, swallowing disorders, and malnutrition that may increase risks of other acute conditions like pneumonia that cause death [2].

Initial assessments are typically done by primary care providers and include a thorough patient history to identify risk factors of AD like family history of dementia and ApoE genotyping where the ε4 allele is associated with increased AD risk [6]. Clinicians must also conduct comprehensive assessments to rule out potential non-AD causes of cognitive impairment like Patient Health Questionnaires [7] for depression, blood tests to assess vitamin, hormone deficiencies, and review of medication side effects. If AD is still suspected, standardized cognitive screening assessments can be conducted before referring to a dementia specialist. The dementia specialist may conduct further assessments including the Jak/Bondi criteria [8], Clinical Dementia Rating (CDR) [9], and National Institute on Aging-Alzheimer's Association criteria [1].

Modern neuroimaging methods provide important insights into the underlying pathophysiologic traits of disease. Structural imaging like MRI can assess for specific patterns of atrophy in the hippocampus and entorhinal cortex where changes are seen early in AD [10]. Regions of increased cortical thickness can also be observed due to compensatory adaptation in networks that are functionally linked to the atrophied areas [11]. The olfactory cortex also shares structural and functional connections with these areas first affected by AD; therefore, subtle changes in olfactory perception can also serve as a clinical indicator of cognitive dysfunction [12]. The Alzheimer’s Association and the Amyloid Imaging Task Force recommend amyloid PET scan to support the diagnosis of AD in concordance with other clinical assessments [4]. Amyloid PET uses tracers (florbetapir, flutemetamol, and florbetaben) that bind specifically to Aβ within plaques. A positive amyloid PET scan will show increased cortical retention of the tracers, thus confirming the presence of Aβ plaques in the brain, indicating AD pathology. Patients can also undergo a lumbar puncture to analyze CSF proteins: Aβ42, phosphorylated tau (p-tau) and total tau (t-tau), which can be used as biomarkers to confirm Aβ plaque presence in the brain. Decreased CSF Aβ42 levels indicate increased Aβ aggregation and deposition in the brain. Specialists can also analyze the concentration of Aβ42 vs. the total amount of Aβ peptides to better index a patient’s pathology. P-tau in CSF is a direct measure of phosphorylated tau in the brain. Increased p-tau levels indicate the presence of NFTs. T-tau in CSF can be used to predict the level of neurodegeneration in a patient with suspected AD [3]. More recently, blood-based biomarkers like plasma p-tau have shown significant promise in identifying AD pathology without the high costs or invasiveness of PET and CSF analysis allowing more scalable options for early detection [10].

Artificial intelligence

Despite the recent excitement surrounding artificial intelligence (AI), this technology has been decades in the making, dating back to the 1960s, when Arthur Samuel of IBM (Armonk, NY, USA) created a checkers computer game that was able to use pattern recognition to reinforce its own learning through experience. Over time, AI has evolved from a rule-based system to machine learning (ML) where algorithms learn from input data to make decisions without the need for manual programming. ML algorithms can be divided into several subgroups like support vector machines (SVM), random forest (RF), and Naive Bayes (NB) based on what type of data it is best suited for [13]. For example, RF is best suited for classification tasks where it creates many decision trees, all individually trained on a random subset of data to combine their results through majority voting across all trees [14].

More recent advancements in graphics processing units like those created by NVIDIA Corp. (Santa Clara, CA, USA) have driven major improvements in parallel computing power and allowed a subset of ML called deep learning (DL) to reach new benchmarks. DL utilizes many (>20) layers of neural networks, automatic feature extraction, end-to-end learning that achieves both better performance and is scalable with big data. A popular DL algorithm is convolutional neural network (CNN) designed for image processing. CNN uses kernels (small filters) that slide across an image, creating an output value based on specific patterns like edges, corners, and textures to automatically learn, discriminate, and identify important features with minimal manual intervention. This allows assortment and analyzation of millions of individual variables to expose underlying patterns. CNN has become essential in modern computing visual systems like facial recognition and autonomous vehicles [14]. Applications of DL have also been successfully employed in medical breakthroughs from the human genome project to diagnosing diabetic retinopathy through smartphone cameras [15]. ML and DL approaches are also being utilized to detect common brain diseases like Parkinson’s and epilepsy. Motion sensors capturing hand movement and calculations based on speed, amplitude, and frequency were used to train ML models to detect early Parkinson's disease at the second and third stages with an SVM classifier achieving 98.4% accuracy. The creation of an application for automatic spike detection in patients with epilepsy was possible because of DL algorithms learned epileptic zones from magnetoencephalography data [16].

Although various hypotheses have been proposed to explain the cause of AD, the exact cause remains unknown. Amyloid PET scans and CSF biomarkers showed that specific AD pathological changes were found to have initiated as early as two decades before the onset of symptoms. Therefore, the diagnosis of AD when symptoms are already present would not be very helpful as pathological changes would have already progressed to higher stages [3]. The rate of disease progression has important implications in clinical practice and in determination of prognosis. About 5%-25% of individuals with MCI will progress to AD per year, while about 16%-23% revert from MCI to cognitive function (CN) [17]. Although there is no cure for AD, therapies are available to slow down disease progression and improve its management [10]. Considering limitations of current diagnostic methods, AI technology can be exploited as a triage, progression risk, and clinical decision support tool to aid clinicians to detect AD earlier and predict disease progression, thereby allowing patients to receive treatment sooner, participate in promising drug trials, and plan for the future. This review aims to explore the state of AI utility in detecting AD early and predict disease progression using clinical trials that integrate ML and DL models across multimodal datasets.

## Review

Methods

Search Strategy and Eligibility Criteria

A systematic literature search was conducted in April 2026 using the PubMed database. The search strategy utilized the Medical Subject Headings (MeSH): ("Alzheimer Disease"[MeSH]) AND ("Deep Learning" OR "Machine Learning" OR "Artificial Intelligence"). Results were filtered to be clinical trials or randomized controlled trials, yielding 36 results. The inclusion criteria consisted of studies that used AI to detect AD pathology in MCI patients or predict MCI to AD progression.

Study Selection and Data Extraction

**Figure 1:**
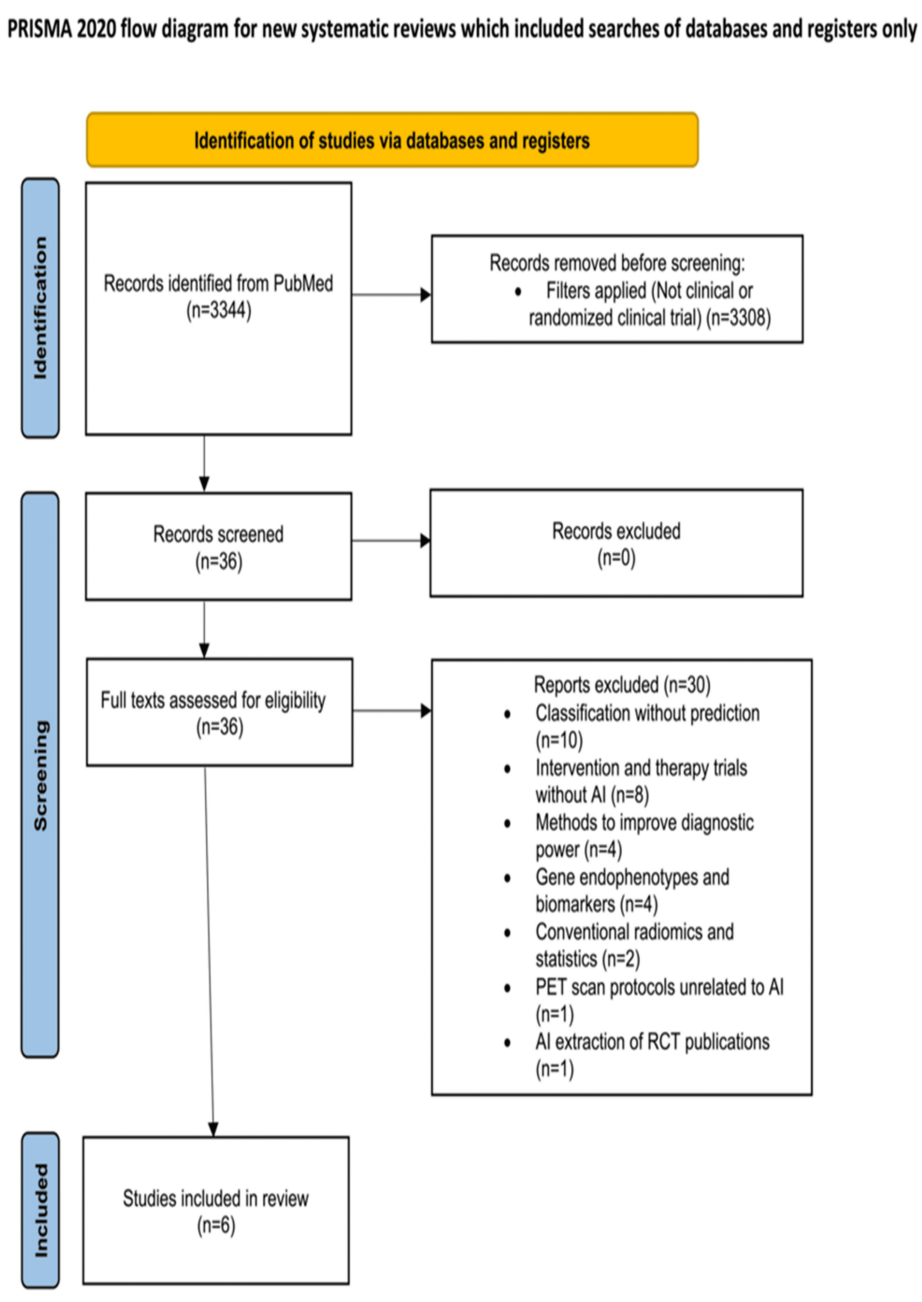
PRISMA Flow Diagram for Study Selection Records were identified from PubMed, screened for eligibility, and six studies were included in the final qualitative review. The flow diagram was adapted from the PRISMA 2020 template. AI: artificial intelligence. PRISMA: Preferred Reporting Items for Systematic Reviews and Meta-Analyses, RCT: randomized controlled trial.

Two independent reviewers screened the titles and abstracts. Disagreements were resolved via consensus. Of the 36 papers screened, 10 papers were excluded for using AI to distinguish existing AD from controls (or MCI from AD) without any prediction or early detection component. Eight were excluded for studying interventions and therapeutics in AD and MCI without using AI prediction models. Four papers focused on methods to improve diagnostic accuracy and study power rather than clinical prediction. Four were excluded for identifying gene endophenotypes and biomarkers linked to distinct AD subgroups. Two studied early AD prediction using conventional radiomics and statistics without an AI model. One was a validation study for a PET scan protocol unrelated to AI. One developed a model for extracting information from randomized controlled trial publications unrelated to AD progression. Finally, six studies met these criteria and were retained for detailed analysis. A formal meta-analysis was not performed because of high methodological and data heterogeneity among the included studies. Figure 1 shows the Preferred Reporting Items for Systematic Reviews and Meta-Analyses (PRISMA)-style flowchart for data selection.

Risk of Bias Assessment

The Quality Assessment of Diagnostic Accuracy Studies 2 (QUADAS-2) tool [18] was selected to evaluate the risk of bias because the included studies focused on diagnostic accuracy and prediction modeling rather than therapeutic interventions. Each study was independently assessed across four key domains: patient selection, index test, reference standard, and flow and timing (Table 1).

**Table 1:** QUADAS-2 Risk of Bias Assessment for Included Studies *The article by Yu et al. [21] (2022) was marked as high risk for patient selection bias because of its small sample size (n=30), which may not reflect the broader clinical population. QUADAS-2: Quality Assessment of Diagnostic Accuracy Studies 2.

Study	Patient Selection	Index Test	Reference Standard	Flow and Timing	Overall Risk of Bias
Kim et al. [19], (2022)	Low	Low	Low	Low	Low
Kim et al. [20], (2023)	Low	Low	Low	Low	Low
Lee et al. [17], (2018)	Low	Low	Low	Low	Low
Yu et al. [21], (2022)	High*	Low	Low	Low	Moderate
Zhao et al. [25], (2022)	Low	Low	Low	Low	Low
El-Sappagh et al. [27], (2021)	Low	Low	Low	Low	Low

Results

Study Characteristics

A total of six studies were included. The methodologies varied widely, encompassing functional near-infrared spectroscopy (fNIRS), SERS analysis of CSF, MRI cortical thickness mapping, and multimodal integration of serum, imaging, and cognitive data. Table 2 summarizes the primary findings and statistical outcomes.

**Table 2:** Evidence Table CN: cognitively normal, MCI: mild cognitive impairment, AD: Alzheimer's disease, fNIRS: functional near-infrared spectroscopy, RF: random forest, ML: machine learning, AUROC: area under the receiver operating characteristic curve, aMCI: amnestic mild cognitive impairment, CSF: cerebrospinal fluid, CNN: convolutional neural network, FA: fractional anisotropy, ADNI: Alzheimer's disease neuroimaging initiative, SHAP: Shapley Additive exPlanations, FAD+: familial Alzheimer's disease positive, FAD-: familial Alzheimer's disease negative, HCA: hierarchical clustering analysis, ASM: Atrophy Similarity Metric.

First Author and Year	Study Design	Level of Evidence	Study Population	Therapy or Exposure	Primary Outcome/Results	Key Statistical Findings
Kim [19], 2022	Patient-level diagnostic trial	Level 3	168 final participants aged ≥60 years (70 CN, 42 MCI due to AD, 21 AD, 35 moderate dementia)	Five scents presented during fNIRS olfactory measurement.	RF test model achieved 96% accuracy, 95% sensitivity, 96% specificity.	AUROC: 90.7% (AD + MCI vs CN), 90.99% (MCI alone), 93.34% (AD alone).
Kim [20], 2023	Post hoc analysis of diagnostic trial with external validation	Level 3	97 participants from original trial, 36 new participants (71 CN, 41 MCI, 21 AD)	Five scents presented during fNIRS olfactory measurement; ensemble stacking ML.	MCI vs CN: ML achieved 81.8% specificity, 88.7% sensitivity in original trial.	MCI vs CN feature importance: fNIRS (1.000), age (0.721), sex (0.710). MCI-AD vs CN AUC: 0.925 (original), 0.825 (validation).
Lee [17], 2018	Patient-level diagnostic trial	Level 3	Cross-sectional cohort: 869 CN, 473 AD. Longitudinal: 70 aMCI, 27 AD	MRI cortical thickness used to compute AD-specific atrophy similarity metric (ASM).	ML achieved 91.1% accuracy, 83.5% sensitivity 95.2% specificity.	Higher ASM in aMCI predicted AD conversion (p<0.001). Higher ASM in AD predicted faster decline (p<0.05).
Yu [21], 2022	Experimental diagnostic performance study	Level 3	30 CSF samples (10 healthy, 9 AD, 5 FAD+, 4 FAD-)	HCA and CNN trained on Raman spectral data from CSF to classify disease state.	HCA classified 97.7% FAD and 93.3% AD correctly. CNN achieved 94% overall accuracy.	CNN accuracy breakdown: 100% healthy, 88.9% AD, 100% FAD+, 80% FAD-.
Zhao [25], 2022	Longitudinal follow-up with ML predictive modeling	Level 3	105 MCI patients aged ≥65 followed for two years	Multimodal dataset comparing stable MCI vs deteriorative MCI.	Model 3 (four features) achieved 100% training accuracy, 97% test accuracy.	Model 3 achieved 100% sensitivity, 95% specificity, 0.99 AUC. FA model alone had 56% test accuracy.
El-Sappagh [27], 2021	Retrospective diagnostic validation study	Level 3	1,048 subjects from ADNI dataset: 294 CN, 254 stable MCI, 232 progressive MCI, 268 AD	Two-layer RF model and SHAP feature attribution trained to integrate 11 data modalities.	RF ensemble achieved 93.33% MCA in first layer. RF achieved 87.76% accuracy in second layer.	First layer AUROC: 90.7% (AD + MCI vs CN). Second layer CV achieved 87.08% AUC; MS2 test set achieved 0.963 AUC.

Early Detection Models

In a prospective, patient-level, single-group, diagnostic accuracy study, Kim et al. [19] explored using RF algorithms to utilize differences in oxygenation levels in the right and left prefrontal cortices as clinical indicators of AD pathology. The study included 178 volunteers aged ≥60 years in Gwangju Metropolitan City in South Korea from March 2, 2021 to March 1, 2022. Six were excluded for refusal of cognitive function tests, and four were excluded because of nasal obstruction or acute respiratory disease. One hundred sixty-eight participants were included in the final sample: 70 had normal cognitive function (CN) (73.9 ± 5.7 years), 42 had MCI due to AD (73.0 ± 6.0 years), 21 had AD (75.6 ± 7.6 years), and 35 had moderate dementia (83.6 ± 5.4 years). Mini-Mental State Examination (MMSE) and Seoul Neuropsychological Screening Battery were performed for cognitive function tests. The MCI due to the AD group had a standardized uptake value of 1.1 or higher on PET CT for Aβ plaques and met the comprehensive Jak/Bondi classification criteria for MCI. Patients in the AD group were diagnosed with AD at a hospital and had a clinical dementia grade of 1 or higher [19].

The total experiment duration was approximately five minutes. Participants were asked to smell five scents (unscented, Downy, peppermint, leather, and a second unscented) with a 40-second break between each scent stimulus. The scents were presented on a sniff stick 5-10 cm away from the nose for 20 seconds. They were allowed to smell naturally, without closing their eyes or blocking one nostril. fNIRS was used to measure differences in oxygenation levels during the sniff tests. The fNIRS probe was placed between the eyebrows to track both oxygenated and deoxygenated hemoglobin in the orbitofrontal cortex by delivering near-infrared wavelengths of 730 and 850 nm. To reduce interference from physiological sources like skin, cardiac, and respiratory signals, wavelet processing and low-pass filter were applied to remove high frequencies and extract pure brain signal. Concentrations of oxidized (oxygenated) hemoglobin and reduced (deoxygenated) hemoglobin were calculated using the modified Beer-Lambert law to obtain the corrected near-infrared spectroscopy (C-NIRS) data. The reduced hemoglobin signals were then subtracted from the C-NIRS oxidized hemoglobin signals to calculate and graph the relative oxygenation difference or oxygen consumption between the left and right orbitofrontal cortex (LR oxygenation difference). The LR oxygenation difference signals represented asymmetric prefrontal cortical activation during olfactory simulation and, therefore, served as a biomarker for AD in the RF model [19].

Researchers built an ensemble RF algorithm, a type of supervised ML, commonly used for classification tasks. To reduce the role of chance, an average of 10 hyperparameters were applied to prevent overfitting and increase generalizability. The best performing training dataset model reported 96% accuracy, 95% sensitivity, 96% specificity, 95% recall, and 95% F1 score. The independent test dataset reported 94% accuracy, 90% sensitivity, 100% specificity, 94% recall, and 93% F1 score. The area under-receiver operating characteristic curve (AUROC) and the area under precision-recall curve reported 90.7% and 84.6% in classifying AD and MCI from CN, 90.99% and 90.98% in identifying MCI alone, and 93.34% and 92.79% in identifying AD alone, respectively. A statistical power analysis determined a minimum of 17 participants were needed per group to achieve 90% statistical power at a 10% significance level. The final group exceeded this requirement by consisting of 70 CN, 42 MCI and 56 AD, running 10 trials with a test size of 34 participants and using a two-sided p-value below 0.05 to monitor statistical significance [19].

Kim et al. [20] built upon the same line of research of the previous study [19] and added an external validation trial to increase reproducibility. A post hoc analysis of the 2022 paper was done where 97 participants from the original 168 were selected. Poor-quality fNIRS, diagnostic data, and those with moderate dementia were excluded. The new independent external validation trial consisted of 36 new participants from the same clinic with the same inclusion criteria but were entirely independent from the original 168. Of the 133 total participants, 71 were CN, 41 had MCI, and 21 had AD dementia. They underwent the identical experiment between July 22, 2022 and August 30, 2022, as the original trial. Researchers developed two ML models and a fivefold cross-validation model to study the classification of MCI vs CN and MCI-AD dementia vs CN. The MCI vs CN utilized an ensemble XGBoost, boost gradient (BG), and light boost gradient (LBG) models. The MCI-AD dementia vs CN utilized an ensemble of two BG and LBG. Results of the ML models were compared to a classical statistical analysis using R software, version 3.1.1 (R Foundation for Statistical Computing, Vienna, Austria) and SPSS (IBM Corp., Armonk, NY, USA) with two-tailed p-values <0.05 considered statistically significant and C-statistics used to show AUC using 95% confidence intervals. A calibration procedure was also performed to the fNIRS data to match values and ensure fair comparison between the statistical and ML models [20].

In the MCI vs CN trial, the ML model outperformed in both datasets achieving 81.8% specificity and 88.7% sensitivity in the original trial and 81.3% specificity and 67.7% sensitivity in the additional validation trial. The classic statistical model achieved 81.8% specificity and 84.6% sensitivity in the original trial and 68.8% specificity and 68.8% sensitivity in the validation trial. In the MCI-AD dementia vs CN trial, the ML achieved 80.0% specificity and 88.1% sensitivity in the original trial and 81.3% specificity and 65.0% sensitivity in the additional validation trial, with an AUC of 0.925 in the original dataset and 0.825 in the external validation dataset. The classic statistical model achieved 81.8% specificity and 88.1% sensitivity in the original trial but dropped to 68.8% specificity and 60.0% sensitivity in the additional trial. In MCI vs CN, feature importance analysis revealed fNIRS having the highest importance value (1.000), followed by age (0.721), sex (0.710), and household income. In the MCI-AD dementia vs CN trial, fNIRS also had the highest importance value followed by sex, age, and smoking status [20].

In an experimental diagnostic performance study, Yu et al. [21] combined surface-enhanced Raman spectroscopy (SERS) data with DL algorithms to detect cerebrospinal fluid (CSF) biomarkers associated with AD. Twenty-eight CSF samples obtained from the University of California, Irvine, Institute for Memory Impairment and Neurologic Disorders, Alzheimer’s Disease Research Center, were categorized into four groups: 10 healthy, nine AD, five familial Alzheimer's disease positive (FAD+), and four familial Alzheimer's disease negative (FAD-). Aβ42, t-tau, p-tau, MMSE, and Clinical Dementia Rating (CDR) scores had already been measured to meet the inclusion criteria. The study was performed in a double-blinded fashion where the sample providers and those performing the spectroscopy and data analysis were all unaware of the sample identities [21].

The SERS platform was based on sphere lithography creating a periodic gold nanopyramid structure. Closely packed monolayer polystyrene balls were spin-coated on a silicon wafer forming a quasi-periodic array with a hexagonal arrangement, and a monolayer of graphene was transferred to the gold-tipped pyramids to enhance Raman signal. The CSF samples were diluted by a factor of 100 before being applied to the SERS platform to keep in the dynamic range of the Raman detection system. A Renishaw inVia Raman microscope (Renishaw plc, Wotton-under-Edge, UK) with 550-1650 cm⁻¹ wavenumber range and Raman mapping with a step size of 3 µm was used under ambient conditions to scan at least 80 spectra per sample. Hierarchical clustering analysis (HCA) was developed using unsupervised statistical methods to cluster similar data points based on similarities and differences. A CNN algorithm was also developed to process and classify all the SERS spectral data. The training dataset which started with 1,200 spectra (minimum 80 per sample × 30 samples) was increased by data augmentation techniques like randomly shifting each spectrum by a few wavenumbers and introduction of random noise. To prevent overfitting and optimize training, early stopping and adaptive gradient algorithm methods were also implemented [21].

HCA studied 15 averaged spectra data from five distinct CSF samples with each set containing three replicates and SERS spectra acquired independently for each replicate. The model grouped the spectra data into five groups of three spectra each. After sample identities were unblinded, replicated data from the same group were found to be consistently grouped together, indicating reproducibility. It was also able to differentiate between all five CSF samples and between individuals with the same disease, indicating that unique CSF biomarkers were identified, and therefore showing sensitivity. HCA classification accuracy for FAD samples (preclinical AD) was 97.7% and 93.3% for clinically diagnosed AD [21].

The CNN model was trained on 17 CSF samples to classify healthy control (HC), AD, FAD+, and FAD- using a “leave-one-group-out” validation strategy. During each evaluation, spectra data from one CSF sample was used for testing, while the rest of the sample data were used for training. This was repeated until every sample had been left out once. The reason for this was to increase generalizability by ensuring that all spectra data in the test set originated from a CSF sample that was not represented in the training set. All predictions during each round of evaluations were combined through a majority vote system which produced a final prediction that achieved an overall 94% diagnostic accuracy. Classification of healthy CSF samples reported 100% accuracy, 88.9% accuracy for AD, 100% accuracy for FAD+, and 80% accuracy for FAD samples. To assess diagnostic validity, researchers conducted a correlation analysis between the CNN-derived prediction index and AD-related medical parameters. It reported high correlation coefficients to three cognitive test scores of r = 0.79 with MMSE, r = -0.88 with Clinical Dementia Rating Sum of Boxes (CDRSB), and r = -0.87 with Clinical Dementia Rating Global Score [21].

Progression Prediction Models

In a multicohort, observational study with both cross-sectional and longitudinal analysis, Lee et al. [17] developed a novel method to predict amnestic mild cognitive impairment (aMCI) progression to AD using ML trained on cortical thickness data. In the cross-sectional cohort, researchers built a custom ML group classifier using principal component analysis (PCA) followed by linear discriminant analysis (LDA) to measure the similarities between AD-specific cortical atrophy patterns of CN and AD patients. This AD-specific cortical atrophy similarity measurement was validated by two longitudinal studies to predict conversion of high-risk aMCI patients progressing to AD and the rate of progression in already diagnosed AD patients [17].

The cross-sectional cohort data used to develop the ML classifier initially included 536 patients with AD dementia and 912 CN. All individuals underwent high-resolution 3T brain MRI with 3D volumetric imaging and detailed neuropsychological testing from the Memory Disorder Clinic of the Samsung Medical Center from June 2006 to June 2012. AD patients satisfied the National Institute of Neurological and Communicative Disorders and Stroke and Alzheimer’s Disease and Related Disorders Association (NINCDS-ARDRA) criteria and had laboratory tests to rule out other causes of dementia. CN individuals had normal cognitive function determined by neuropsychological tests and no history of neurological or psychiatric disorders. After exclusions for unreliable cortical thickness measurements, blurring of MRI images, misclassification of tissue type, inexact surface extraction, severe white matter hyperintensities, and missing education data, the final AD group consisted of 473 patients. A total of 869 CN individuals were included. The aMCI longitudinal cohort testing the validation of AD-specific atrophy similarity scores consisted of 70 aMCI patients who were diagnosed using the Petersen criteria [22], completed at least their first-year follow-up visit and were recruited from August 2007 to December 2010. Fifty-three were labeled non-converters if they were diagnosed with aMCI at baseline and remained so during the first year follow-up. Twenty-six were labelled converters if they were diagnosed with aMCI at baseline and diagnosed with AD during the first year follow-up visit. The AD longitudinal cohort consisted of 27 AD patients who participated in the prospective, five-year longitudinal Alzheimer’s Disease and Positron Emission Tomography study and were recruited from March 2006 to December 2006. They also satisfied the Diagnostic and Statistical Manual of Mental Disorders, Fourth Edition (DSM-IV) [23] and NINCDS-ARDRA criteria [24] for probable AD, CDR score of 0.5 or 1, caregiver availability, and completed the third-year evaluation. Thirteen were grouped as fast-decliners if their Clinical Dementia Rating Sum of Boxes (CDR-SB) score increased more than five points during the three-year follow-up and slow-decliners if their score increased less than five points [17].

Cortical thickness data from MRI scans were preprocessed using FreeSurfer 5.1.0. (Athinoula A. Martinos Center for Biomedical Imaging, Charlestown, MA, USA) to construct each subject’s cortical surface using 40,062 vertices for each hemisphere. It was then filtered using manifold harmonic transform to remove high-frequency noise to minimize artifacts. This noise-filtered cortical thickness data was converted to w-score, adjusting for age and education level to minimize demographic effects on cortical thickness. Cortical atrophy pattern analysis was then quantified using Inbrain software (MIDAS Information Technology Co., Seongnam, South Korea) to train a group classifier and to compute an AD-specific pattern similarity. The classifier was trained by PCA and LDA in sequence to best distinguish AD from CN patterns. PCA is an unsupervised dimensionality reduction ML technique that converted the cortical thickness w-scores into high-dimensional feature vectors while retaining the most variance in the data. LDA is a supervised classification model that found linear combinations of PCA features and learned coordinated axes that maximally separated AD and CN patterns. Ten-fold cross-validation procedure was performed, and participants were randomly separated into 90% training and 10% test sets. After training, an individual’s noise-filtered cortical thickness data was projected into the trained PCA space and then transformed into LDA space using the trained LDA matrix to represent a single point. The AD-specific pattern similarity measurement was calculated by using the Euclidean distance between the subject’s point and the mean point of the AD group in the LDA space. A smaller distance meant a higher similarity score and, therefore, a more similar atrophy pattern of the subject to the representative AD group. A statistical analysis was also done to test the similarity score in relation to meaningful clinical differences over time using student’s t-test and chi-square or Fisher’s exact test with statistical significance set at p<0.005 [17].

In the cross-sectional cohort, AD patients were older than CN (73.0 ± 9.4 vs. 65.4 ± 9.0 years), had fewer years of education (9.4 ± 5.3 vs. 11.7 ± 4.9), more apolipoprotein E epsilon 4 (APOEε4) alleles (55.6% vs. 22.8%), higher prevalence of hypertension (43.6% vs. 29.9%), and lower MMSE scores (18.2 ± 5.5 vs. 28.5 ± 2.0) with p<0.001. In the longitudinal aMCI cohort, converters had more APOEε4 alleles (60.9% vs. 29.4%, p=0.010) and lower baseline MMSE (25.4 ± 3.0 vs. 27.0 ± 1.9, p=0.015) than non-converters. The ML group classifier achieved 91.1% accuracy, 83.5% sensitivity, and 95.2% specificity in discriminating AD from CN, with the entorhinal cortex and precuneus being the most discriminative regions with some contributions from the lateral temporal lobe and prefrontal cortex. In the aMCI cohort, the AD-specific similarity score was higher in converters than non-converters at baseline (p<0.001), first-year (p<0.001), and had greater increases in similarity (β=3.6, standard error SE=1.6, p=0.027). In the AD cohort, fast-decliners also had higher similarity scores than slow-decliners at baseline (p=0.042), first-year (p=0.028), and third-year (p=0.027), with greater increases over time (β=2.9, SE=1.3, p=0.029). Mixed effects models showed AD-specific atrophy similarity by time interactions in MCI patients for language, Seoul Neurophysiological Screening Battery-Dementia version (SNSB-D) total score, and CDR-SB. Significant time interactions in AD patients were found in attention, language, memory, frontal/executive, SNSB-D total, MMSE, CDR, and CDR-SB, all at p<0.05 [17].

In a prospective observational cohort study, Zhao et al. [25] explored using RF algorithms to simultaneously analyze serum and multimodal MRI features to build prediction models for deteriorative MCI due to AD. All subjects were aged 65 years and above recruited from the Heqing community in Shanghai during a free medical examination program conducted by the local government. Volunteers with a history of stroke, Parkinson’s, infections, poisoning, trauma, severe hearing impairment, mental illness, and alcoholism were excluded. Eight physicians, from the Shanghai Mental Health Center, performed the cognitive function assessments, and blood and imaging tests were performed to exclude other causes of MCI. MCI was defined with the presence of cognitive complaints with a CDR score of less than 0.5, objective cognitive impairment in at least one domain based on performance 1.5 SD below the mean using the norms, normal CDR and ADL evaluations, and the absence of dementia using DSM-IV criteria. MCI deterioration was evaluated using the Global Deterioration Scale (GDS) [26] over a two-year follow-up. Scores of ≥1 were assigned to the deterioration group (DG), and those with scores <1 were assigned to the non-deterioration group (NDG) [25].

Demographic data, hematological biomarkers, and multimodal MRI imaging metrics totaling 357 individual features from each subject were collected to be analyzed. The demographic data consisted of age, sex, education years, and history of hypertension and diabetes. Hematological markers consisted of serum Aβ1-40, Aβ1-42, P-tau181, monocyte chemoattractant protein-1 (MCP-1), and APOE gene polymorphisms. MRI images were conducted on a 3.0T system using T1-weighted imaging, diffusion-weighted imaging, diffusion tensor imaging, and arterial spin labeling. The indices selected were fractional anisotropy (FA), cerebral blood flow (CBF), and apparent diffusion coefficient (ADC). The images were skull-stripped and registered to Montreal Neurological Institute space using the SyN method (Advanced Normalization Tools (ANTs), Penn Image Computing and Science Lab, Philadelphia, PA, USA). Automated anatomical labeling parcellation was then employed to extract FA, ADC, and CBF median values from 116 brain regions. Those in the follow-up population with complete data were randomly divided into a 70% training set and 30% test set. Statistical analysis was performed using RF implemented on a Python-based Anaconda platform and recursive feature elimination (RFA) to identify important features with a p-value < 0.05 considered statistically significant [25].

A total of 105 participants completed the two-year follow-up. Forty-one were found to be deteriorative (DG) and 64 non-deteriorative (NDG). After comparison of demographic and hematological baseline characteristics, APOE genotype and age were shown to have significant differences between the two groups. The average age in DG was 72.76 ± 5.35 years, compared to 70.52 ± 4.79 years in NDG (p =0.028). APOE4 was present in 91.4% of DG but only 8.6% in the NDG (p <0.001). There were no statistically significant differences found in serum Aβ1-40, Aβ1-42, P-tau181, or MCP-1 levels between the two groups [25].

Five machine learning models were built using RF and Recursive Feature Elimination (RFE) to compare full and simplified datasets and to find optimal features combinations. Model 1 incorporated all 357 features per subject achieved 100% accuracy on the training set and 64% accuracy on the test set. Model 2 utilized the top five most influential features consisting of APOE4 genotype, FA values of the left fusiform gyrus, left inferior temporal gyrus, left parahippocampal gyrus, and ADC value of the right calcarine fissure. Model 2 achieved 100% accuracy in the training set and 85% accuracy in the test set. Model 3 focused on the top four features, excluding the ADC value of the right calcarine fissure, achieved the best overall performance of 100% accuracy in the training set, 97% accuracy in the test set, with 100% sensitivity, 95% specificity, and AUC of 0.99. Model 4 using the top three features, excluding the FA value of left parahippocampal gyrus, achieved 100% accuracy in the training set and 94% in the test set. Model 5 was based only on hematological indicators including Aβ1-40, Aβ1-42, P-tau, MCP-1, and APOE genotype achieved 100% training accuracy and 91% test accuracy. Control models based solely on demographic or imaging characteristics like FA, CBF, and ADC values performed poorly in comparison; for example, the FA model alone achieved only 56% test accuracy [25].

In a retrospective machine learning study, El-Sappagh et al. [27] developed a two-layer RF model to simultaneously diagnose AD and predict MCI progression to AD. The first layer performs a multi-class classification to distinguish between CN, MCI, and AD subjects based on the whole dataset. The second layer focuses on the MCI cases and conducts a binary classification to predict whether the MCI patient will progress to AD within three years from baseline diagnosis. The multimodal dataset was collected from the Alzheimer’s Disease Neuroimaging Initiative (ADNI) database and consisted of a total of 1,048 subjects with 294 CN, 254 stable MCI (sMCI), 232 progressive MCI (pMCI), and 268 AD. Eleven modalities comprising cognitive scores (CS), neuropsychological battery (NB), MRI images, PET scans, genetics, medical history (MH), lab tests, demographics, vital signs, symptoms, and physical examinations were initially included before feature selection identified the most informative features [27].

Researchers used RFE in combination with RF, SVM, and Gradient Boosting (GB) algorithms to score features and progressively removed the least important ones until the best subset was found. In the first layer, the RF-RFE model achieved the highest accuracy of 94.4% using 28 features (15% of total features) consisting of eight CS, six NB, two MRI features, three PET, five genetics, and four MH features. In the second layer, the RF-RFE model also achieved the highest accuracy of 86.80% using 36 features (19% of total features) consisting of 14 CS, five MRI, three PET, five genetics, and nine MH features. To prevent overfitting, a 10-fold cross-validation procedure was performed where the model was trained on nine parts of the dataset, tested on one, and repeated until each part was tested at least once. One part called the “MS2 test set” was reserved for a final unbiased evaluation and not used for training or cross-validation. Researchers also utilized a three-layered approach to provide justification and explanations for every RF decision to help physicians understand the ML’s predictions in natural language. The three layers included Shapley Additive exPlanations (SHAP) which quantifies how much each feature influenced a given prediction and two post hoc explanations using 11 decision tree (DT) and 11 fuzzy rule-based system (FURIA) explainers [27].

In the first layer examining classification and early AD detection performance, the researchers conducted a set of experiments using the whole training dataset and different combinations of the six selected modalities. Using all modalities in the whole dataset, the RF model achieved a precision of 98.83% for CN, 90.91% for MCI, and 92.41% for AD; recall of 96.21% for CN, 95.45% for MCI, and 86.61% for AD; and multiclass accuracy (MCA) of 93.42% and a multiclass F1 score (MCF) of 93.39%. The best performing subset was the combination of CS, NB, and Genetics. For the MS2 test set, the CS, NB, and Genetics combination achieved a precision of 100.0% CN, 86.79% MCI, and 100.0% AD; and recall of 86.67% CN, 100.0% MCI, and 89.66% AD. It achieved MCA of 93.33% and MCF of 93.82%. The RF ensemble approach outperformed other ML classifiers where RF achieved an 93.33% MCA and 93.82% MCF. SHAP determined the most influential feature was CDR-SB. MMSE was the most predictive for AD, and CDR-SB was the most predictive for CN and MCI classifications. High CDR-SB values had more impact in predicting AD classes. High ADNI_MEM, DigitalTotalScore, and Montreal Cognitive Assessment (MoCA) values were associated with CN. The most widely used explainer was FURIA-based cognitive scores achieving 92.4% coverage indicating the importance of cognitive assessments [27].

In the second layer examining prediction of an sMCI patient progressing to pMCI within three years, the CS+NB, MRI, PET and genetics combination achieved the best performance. In the 10-fold cross-validation (CV) data, it achieved 87.08% accuracy, 88.07% precision, 86.13% recall, 87.09% F1, and 87.08% AUC. For the MS2 test set, the same feature combination set achieved a 91.86% accuracy, 91.70% precision, 91.70% recall, 91.84% F1, and 0.963 AUC. The RF model also outperformed other ML classifiers in the second layer where RF achieved 87.76% accuracy, 87.75% F1, and 0.953 AUC. SHAP determined that the most influential feature was the Functional Activities Questionnaire (FAQ) [28]. MRI features such as hippocampal and intracranial volume and PET features also played a role in progression prediction. The explainers with the highest coverage were achieved by both DT and FURIA-based cognitive scores of 78% also indicating the dominant role of cognitive data in progression predictions [27]. Table 1 shows a summary of the findings of the included studies.

Discussion

While current diagnostic methods rely on post-symptomatic identification of AD pathology, all six studies included in this primary review demonstrated AI has strong potential to transform early detection and prediction of AD deterioration. Although there were some common limitations throughout, each study provided unique methodological techniques where AI consistently achieved higher performance metrics compared to traditional diagnostic and statistical methods. 

Findings

The study by Kim et al. [19] demonstrated RF models achieving excellent predictive performance in detecting AD by analyzing asymmetric cerebral metabolic rates during olfactory stimulation. Kim et al. [20] supplemented this 2022 study by conducting a post hoc and external validation analysis utilizing ensemble ML models that consistently achieved higher diagnostic accuracy than classical statistical models, higher AUC values, and stronger reproducibility across different datasets. Feature importance analysis determined that the fNIRS measurements of cerebral oxygenation differences were the most influential predictor outperforming demographic and clinical variables like age and sex. These findings show this novel olfactory-stimulated fNIRS has physiological relevance and can be used as a quantifiable biomarker for cognitive impairment.

Yu et al. [21] found that their hybrid approach of combining SERS data with DL models was capable of identifying AD pathological signatures in CSF. The CNN prediction index reported a strong correlation between the model’s predicted scores and cognitive test scores indicating that the AI’s prediction correlated with the disease state. Correlation between single biomarkers were relatively low, highlighting that no single biomarker can sufficiently identify AD pathology. The “leave-one-group-out” validation strategy increased generalizability.

Lee et al. [17] demonstrated that their AD-specific atrophy similarity score can serve as an MRI-based biomarker to be used for early detection of AD and prediction of future conversion of aMCI to AD. aMCI participants who converted to AD showed a higher similarity score than non-converters and with greater increases over time. This established that the similarity score was already elevated before clinical conversion and then continued to increase as clinical symptoms worsened. On the other hand, non-converters showed lower and more stable scores, indicating a nonprogressive disease course. Fast-decliners showed higher and steeper longitudinal increases in similarity scores than slow-decliners showing a higher score corresponds with faster deterioration in patients with AD.

Zhao et al. [25] utilized five RF models analyzing 357 features to predict rapidly deteriorative MCI due to AD. They concluded that model 3, which was developed on four features of APOE4 genotype, FA value of left fusiform gyrus, FA value of inferior temporal gyrus, and FA value of parahippocampal gyrus, showed the best predictive effect. They found that it was not necessary to incorporate as many features as possible as it may lead to diminishing returns. This indicated that the four features in model 3 could serve as the four most dominant features contributing to the rapid deterioration form of MCI.

El-Sappagh et al. [27] found that a two-layer RF model integrated on multimodal features and explainers can enhance clinical understanding of AI’s prediction. The RF model outperformed all other ML classifiers with both layers. Explainers like SHAP determined that CDR-SB and MMSE were consistently the most influential predictors in the first layer and FAQ in the second layer. This shows the important role cognitive function measurements plays in clinical diagnosis and prediction of disease progression.

Shared Limitations

Small sample sizes were a common limitation seen across most studies. Kim et al. [19] originally had 178 volunteers but 42 with MCI and 56 with AD were ultimately analyzed after data exclusions. In the follow-up study, Kim et al. [20] had only 36 new participants for external validation which although increases reproducibility, is a restriction on generalizability and statistical power. Yu et al. [21] had 30 total CSF samples, and Zhao et al. [25] had only 105 patients that followed up. Small datasets increase risk of overfitting in ML where there is too little data for models to recognize generalizable patterns. This is seen in Zhao et al. [25] where 357 total features trained on a small sample of 105 participants created a high feature-to-sample ratio. This resulted in the RF achieving 100% accuracy in the training set. Although this may seem ideal, it is actually the result of overfitting and the model finding illegitimate correlations that only exist in the training set. The model may fail when new data is added. Some studies also had unequal distribution of datasets that cause potential bias. Kim et al. [19] had 70 CN, 42 MCI, and 56 AD. This imbalance can cause higher sensitivity for the larger groups and decrease accuracy for the smaller groups and, therefore, diminish power.

Most studies sourced datasets from a single site or geographic region contributing to further concern about generalizability. Three studies were conducted in South Korea, one in China, and one in the U.S, and the dataset by El-Sappagh et al. [27] was solely derived from ADNI. This is a limitation because a lack of diverse data and external validation can miss certain cultural, environmental, or genetic influences. An example is that the APOE4 allele frequency can vary among certain ethnic groups and, therefore, can play an unintended role in the outcome of the study if all the data were derived from a single ethnically homogenously geographical location. Kim et al. [20] was the only study to conduct an external validation trial by testing their fNIRS model on 36 new participants. However, this new cohort came from the same institution with the same ethnic population, therefore, limits generalizability because of lack of heterogeneity of real-world populations.

Another limitation was the lack of longitudinal follow-up. AD is a progressive disease in which disease pathophysiology can begin more than a decade before symptom onset; therefore, assessment of model performance over time is important in evaluating its reliability. Zhao et al. [25] had two-year follow-up design, and Lee et al. [17] monitored the atrophy similarity score over a three-year period. These follow-ups are still relatively short in the scope of AD progression and may capture only transient patterns rather than stable, disease-defining biomarkers that are important in predicting disease evolution through decades. The other studies utilized cross-sectional datasets to classify participants at a single time point and therefore cannot be relied on to predict disease progression.

Another limitation was the lack of model interpretability. Five of the six studies provided little insight into the AI’s decision-making process and, instead, focused on accuracy rather than clinical interpretability. Only El-Sappagh et al. [27] included explainers like SHAP and FURIA to provide justifications for each prediction using natural language to help physicians understand how an individual feature influenced the final prediction. This brought transparency and increased trustworthiness of the model’s processes because it allows physicians to relate AI decisions to known disease mechanisms.

The final limitation is a methodological limitation. The review relied only on a PubMed-only search strategy. Many AI, ML, neuroimaging, and computational biomarker studies are published outside traditional clinical indexing; so, the review may have excluded important relevant studies. Future reviews should incorporate engineering databases such as Embase, Web of Science, IEEE Xplore, ACM Digital Library, and Scopus to be more representative of current AI literature.

Methodological Advances and Future Recommendations

Each study offers unique methodological techniques highlighting the diverse range of AI applications in AD. Kim et al. [19,20] introduced a noninvasive, low-risk biomarker utilizing fNIRS technology. Yu et al. [21] explored combining SERS with DL algorithms to associate biochemical changes in CSF to disease state. Lee et al. [17] created an AD-specific atrophy similarity score using AI to serve as an imaging biomarker to predict AD progression. Zhao et al. [25] compared several RF models to identify key features that best signal early cognitive decline due to AD. El-Sappagh et al. [27] proposed a clinically applicable AI model that can bridge the gap between ML performance and clinical trustworthiness. Future AI studies must also adhere to standardized reporting and bias assessment frameworks like Transparent Reporting of a multivariable prediction model for Individual Prognosis or Diagnosis (TRIPOD)+AI [29] for predictive models, Prediction model Risk Of Bias Assessment Tool (PROBAST)+AI [30], and QUADAS-AI [31]. These tools work well with AI-specific concerns like data leakage, missing data handling, and clinical calibration, which enhances model usability in real-world triage or decision support scenarios.

Because AI technology is still relatively new and each approach was novel in its own way, the lack of a standardized approach makes it difficult for independent validation. For example, each study consisted of their own biomarker and their own data preprocessing methods. This intermixture of different input modalities, feature selection methods, and data preprocessing puts a restriction on reproducibility. Future research should create a unified multimodal framework that takes into account AD’s multifactorial pathophysiology utilizing AI’s ability to model and integrate complex interactions. This can allow for implementation of large, multi-center, multi-ethnic, longitudinal, and external validation collaborations across independent datasets to improve predictive accuracy and generalizability. Future studies should also include interpretability techniques essential for clinical adoption as clinicians should understand why a model made its prediction. They should also consider cost-effectiveness and patient ethics in which whether early detection reduces downstream healthcare costs or improves patient outcomes.

## Conclusions

Overall, the research reviewed here demonstrates that AI techniques show strong potential and promise in detecting early AD pathology and predicting the progression of MCI. ML and DL algorithms leveraged diverse data modalities including neuroimaging, CSF biomarkers, neuropsychological assessments, and physiological signals to consistently outperform conventional statistical approaches in sensitivity and specificity. However, translating these models into clinical practice requires overcoming several challenges related to small sample sizes, demographic homogeneity, and algorithmic transparency. Future research should aim to use larger, multi-center, longitudinal trials that integrate multimodal datasets and explainable AI to enhance transparency and build clinical trust.
